# Supplementary material for: Imaging single-cell blood flow in the smallest to largest vessels in the living retina
Source: eLife. 2019 May 14;8:e45077. doi: 10.7554/eLife.45077 (PMC6516827; doi:10.7554/eLife.45077)
Supplement: Supplementary file 1. — Our study is focused on (1) the method and (2) generating a novel dataset of hemodynamics in the healthy mouse. As such, Figures 1–9 are designed to show the method and analysis steps, algorithm validation and representative examples of measurements made. Therefore, these figures typically show one or a few vessels, from one mouse, unless otherwise mentioned in the table above. The population data across all mice imaged is shown in Figure 10. Population data are reported for mean velocity, flow, diameter and flux, across multiple mice, as quantified in the table above. [file elife-45077-supp1.pdf]

Number and Type of Mice Used in Each Figure

Figure Number	Species	No. of mice used	Age (weeks)	Sex	Weight (g)	Animal ID	Eye	Date imaged (MM-DD-YYYY)
1	C57BL/6J	1	31	Male	43	01325	Right	09-27-2017
2	C57BL/6J	1	31	Male	43	01325	Right	09-27-2017
3	C57BL/6J	1	-	-	-	-	Left	09-25-2014
4	C57BL/6J	1	31	Male	43	01325	Right	09-27-2017
4 – supplement 1 A	C57BL/6J	1	31	Male	43	01325	Right	09-27-2017
4 – supplement 1 B,C	C57BL/6J	1	15	Male	26.5	01346	Right	09-28-2017
5 A,B	C57BL/6J	1	31	Male	43	01325	Right	09-27-2017
5 C	C57BL/6J	1	-	-	-	-	Left	09-25-2014
6	C57BL/6J	1	31	Male	43	01325	Right	09-27-2017
7	C57BL/6J	1	37	Male	42.5	01325	Right	11-08-2017
8	C57BL/6J	1	-	-	-	-	Left	09-25-2014
9	C57BL/6J	1	15	Male	26.5	01346	Right	09-28-2017
10 A,B (without capillaries)	C57BL/6J	9	15-73	7 Males<br>1 Female<br>1 unknown	39.1 ± 6.5	NA	NA	09-25-2014 to 11-08-2017
10 C-F (only capillaries)	C57BL/6J	10	13-37	4 Males<br>3 Female<br>3 unknown	26.6 ± 4.0	NA	NA	03-31-2015 to 01-14-2016
**Complete paper (i.e. Fig. 10)**	C57BL/6J	19	13-73	11 Males<br>4 Females<br>4 unknown	31.7 ± 8.1 (mean ± SD)	NA	NA	09-25-2014 to 11-08-2017

Supplementary Table 1: The number and type of mice used in each figure. Our study is focused on 1) the method and 2) generating a novel dataset of hemodynamics in the healthy mouse. As such, Figures 1 to 9 are designed to show the method and analysis steps, algorithm validation and representative examples of measurements made. Therefore, these figures typically show one or a few vessels, from one mouse, unless otherwise mentioned in the table above. The population data across all mice imaged is shown in Figure 10. Population data are reported for mean velocity, flow, diameter and flux, across multiple mice, as quantified in the table above.
