# Supplementary material for: Imaging single-cell blood flow in the smallest to largest vessels in the living retina
Source: eLife. 2019 May 14;8:e45077. doi: 10.7554/eLife.45077 (PMC6516827; doi:10.7554/eLife.45077)
Supplement: Transparent reporting form [file elife-45077-transrepform.pdf]

eLIFE
1st Floor
24 Hills Road
Cambridge CB2 1JP, UK
P 01223 855340
W elifesciences.org
T @elife

eLife's transparent reporting form

We encourage authors to provide detailed information *within their submission* to facilitate the interpretation and replication of experiments. Authors can upload supporting documentation to indicate the use of appropriate reporting guidelines for health-related research (see EQUATOR Network), life science research (see the BioSharing Information Resource), or the ARRIVE guidelines for reporting work involving animal research. Where applicable, authors should refer to any relevant reporting standards documents in this form.

If you have any questions, please consult our Journal Policies and/or contact us: editorial@elifesciences.org.

Sample-size estimation

- You should state whether an appropriate sample size was computed when the study was being designed
- You should state the statistical method of sample size computation and any required assumptions
- If no explicit power analysis was used, you should describe how you decided what sample (replicate) size (number) to use

Please outline where this information can be found within the submission (e.g., sections or figure legends), or explain why this information doesn't apply to your submission:

This information can be found in the following manuscript sections:

Number of mice: Methods – Animals
Number and types of vessels imaged: Methods – Types of blood vessels studied

These reported measures are the first of their kind, establishing a normative database in the full spectrum of mouse retinal vessel sizes. Therefore, no power analysis was available to instruct sample size. To provide a baseline for future power analysis, 19 C57BL/6J mice (postnatal weeks 13-73)were imaged constituting 123 vessels imaged.

To provide further power and specificity for future study, vessels were categorized by vessel generation (branch order relative to disc), vessel diameter (measured objectively with AOSLO) and type (arteriole/venule/capillary) based on direction of flow and lumen diameter.

Unless otherwise described, data is reported as population standard deviation relative to mean. Where compared across mice, standard deviation represents velocity variability across the group (e.g. vessels in different mice of the same vessel generation). When compared across time (e.g. when compared across multiple cardiac cycles in the same vessel, and in the same mouse), variability is represented as standard deviation across sequential cardiac cycles.

Replicates

- You should report how often each experiment was performed
- You should include a definition of biological versus technical replication
- The data obtained should be provided and sufficient information should be provided to indicate the number of independent biological and/or technical replicates
- If you encountered any outliers, you should describe how these were handled
- Criteria for exclusion/inclusion of data should be clearly stated

- High-throughput sequence data should be uploaded before submission, with a private link for reviewers provided (these are available from both GEO and ArrayExpress)

Please outline where this information can be found within the submission (e.g., sections or figure legends), or explain why this information doesn't apply to your submission:

Experiments were performed from dates 2014 to 2017 using a single custom built AOSLO tailored for the mouse eye. 123 vessels were imaged over this time, system did not undergo modifications that would alter data collection during that time and was routinely calibrated and aligned by the authors on a weekly to monthly basis.

Biological replication was investigated in several ways:
1) by examining blood velocity in the same vascular branch within the same mouse. Conservation of flow served as a measure that independently confirmed that blood velocity scaled by lumen diameter provides vessel flow. Accounting for branch order, divisions in flow matched within 8.9% of the predicted value of two diverging branches (addressed in figure 9).
2) Thousands of repeat measures were performed sequentially in the same vessel for 10 seconds allowing serial measures of flow variability as a function of cardiac cycle (figure 4). We observe similar but not identical velocity measures as a function of each heart beat. This reveals subtle differences in true biological replication of blood velocity.
3) Independent measures across the same vascular lumen revealed distinct velocities as a function of distance of blood cell relative to the vascular wall. This data which is objectively measured is similar to the known properties of fluid flow in tube networks (figure 6).

Technical replicates were conducted on the blood velocity software in two ways:
1) the Radon code revealed the same velocity profile when run on the same data set multiple times (data not shown)
2) the code solved the correct local angle as confirmed with a ground-truth data set where local angles were known a priori. (data can be provided, but will be published in a separate technical publication that focuses on a distributable and executable code beyond the scope of this paper)

Outlier handling:
Data from all mice were included. All vessels captured from each mouse was included.
-Algorithmically, conditions of velocity determination are described in methods which provides limits on velocity bandwidth based on spatio-temporal resolution, angle search space

Statistical reporting

- Statistical analysis methods should be described and justified
- Raw data should be presented in figures whenever informative to do so (typically when N per group is less than 10)
- For each experiment, you should identify the statistical tests used, exact values of N, definitions of center, methods of multiple test correction, and dispersion and precision measures (e.g., mean, median, SD, SEM, confidence intervals; and, for the major substantive results, a measure of effect size (e.g., Pearson's r, Cohen's d)

eLife Sciences Publications, Ltd is a limited liability non-profit non-stock corporation incorporated in the State of Delaware, USA, with company number 5030732, and is registered in the UK with company number FC030576 and branch number BR015634 at the address 1st Floor, 24 Hills Road, Cambridge CB2 1JP | August 2014

- Report exact p-values wherever possible alongside the summary statistics and 95% confidence intervals. These should be reported for all key questions and not only when the p-value is less than 0.05.

Please outline where this information can be found within the submission (e.g., sections or figure legends), or explain why this information doesn't apply to your submission:

This information can be found in the methods section where number of samples, mice, and repeat measures are described.

Statistical analysis: Methods – Statistics Table 1

(For large datasets, or papers with a very large number of statistical tests, you may upload a single table file with tests, Ns, etc., with reference to sections in the manuscript.)

Group allocation

- Indicate how samples were allocated into experimental groups (in the case of clinical studies, please specify allocation to treatment method); if randomization was used, please also state if restricted randomization was applied
- Indicate if masking was used during group allocation, data collection and/or data analysis

Please outline where this information can be found within the submission (e.g., sections or figure legends), or explain why this information doesn't apply to your submission:

This manuscript describes an automated computer algorithm. The velocity algorithm is agnostic to the vessel lumen diameter therefore it cannot introduce subjective bias in velocity determination. This is a benefit from human subjective measurements which may introduce bias and has been previously reported (see first paragraph of discussion).

As per above, masking was not performed.

As described above, all data was used without rejection of vessel outliers. Vessels were grouped based on their diameter and type (arteriole/venule). Diameter was measured objectively. Vessel type was indicated by direction of flow (arteriole: from the disc, venule: to the disc.)

Additional data files ("source data")

- We encourage you to upload relevant additional data files, such as numerical data that are represented as a graph in a figure, or as a summary table
- Where provided, these should be in the most useful format, and they can be uploaded as "Source data" files linked to a main figure or table
- Include model definition files including the full list of parameters used
- Include code used for data analysis (e.g., R, MatLab)
- Avoid stating that data files are "available upon request"

Please indicate the figures or tables for which source data files have been provided:

-Matlab code is provided
-global and local variables are contained within
-raw AOSLO data is large in size, constituting 100s of GBs of data. One representative file is provided so that users can see raw data format and resolution (see video 2 included)
